# Supplementary material for: Phytochemical Investigation and Biological Studies on Selected Searsia Species
Source: Plants (Basel). 2022 Oct 21;11(20):2793. doi: 10.3390/plants11202793 (PMC9606921; doi:10.3390/plants11202793)
Supplement: Supplementary file 1 [file plants-11-02793-s001.zip › plants-1938657 supplementary.pdf]

Supplementary Material

Phytochemical Investigation and Biological Studies on Selected *Searsia* Species.

Mkhuseli Koki [1], Masande Yalo [1,2], Masixole Makhaba [1,3], Ndikho Nako [1], Fanie Rautenbach [4], Jelili A. Badmus [4], Jeanine Marnewick [4] Ahmed A. Hussein [3,*], Wilfred T. Mabusela [1,*]

[1] Department of Chemistry, University of the Western Cape, Private Bag X17, Bellville 7538, South Africa

[2] Department of Pharmacology, Faculty of Health Sciences, University of Free State, Bloemfontein, 9300, South Africa.

[3] Chemistry Department, Cape Peninsula University of Technology, Symphony Rd., Bellville 7535, South Africa

[4] Applied Microbial and Health Biotechnology Institute, Cape Peninsula University of Technology, Symphony Rd., Bellville 7535, South Africa

* Correspondence: Correspondence: mohammedam@cput.ac.za (A.A.H.); wmabusela@uwc.ac.za (W.T.M.);
Tel.: +27-(0)-21-959-6193 (A.A.H.); +27-(0)-21-959-3052 (W.T.M.);
Fax: +27-(0)-21-959-1281 (W.T.M.)

Moronic acid (1) and 21β-hydroxylolean-12-en-3-one (2)

Table S1: ^{1}H (400 MHZ) and ^{13}C (100 MHz) NMR spectral data of **C1** and **C2** in $CDCl_3$

	C1		C2	
**No.**	**δ_H (*mult, J*)**	**δ_C**	**δ_H (*mult, J*)**	**δ_C**
**1**	1.98 (*m*)	39.8		39.9
**2**	2.40 (*m*)	33.8	1.90 (*m*)	34.2
**3**		218.3	2.41 (*m*)	217.8
**4**		47.3		47.6
**5**	1.39(*m*)	55.3		55.3
**6**	1.38 (*m*)	19.6	1.33 (*m*)	19.6
**7**	2.49 (*m*)	33.9	1.51 (*m*)	33.8
**8**		40.6	2.37 (*m*)	41.3
**9**	1.39 (*m*)	50.5		50.6
**10**		36.8	1.34 (*m*)	37.1
**11**	1.33 (*m*)	21.4		20.1
**12**	1.28 (*m*)	26.0	1.46 (*m*)	122.4
**13**	2.27(*m*)	41.6	5.33 *t*, *J* = 7.08 Hz	143.6
**14**		42.6		41.7
**15**	1.27 (*m*)	29.3		29.7
**16**	1.48 (*m*)	33.7	1.27 (*m*)	29.6
**17**		47.9	1.36 (*m*)	36.7
**18**		136.6		46.8
**19**	5.19 (*s*)	132.4	0.78 (*m*)	45.8
**20**		32.1	1.19 (*m*)	36.7
**21**	2.22 (*t*)	33.4		78.9
**22**	2.19 (*t*)	33.3	3.22 *dd*, *J* = 11,2; 5.3 Hz)	45.2
**23**	1.05 (3H, *s*)	20.9	1.19 (*m*)	23.6
**24**	1.09 (3H, *s*)	26.8	0.96 3H, (*s*)	26.4
**25**	1.04 (3H, *s*)	15.8	0.86 (3H, *s*)	15.4
**26**	0.97 (3H, *s*)	16.7	0.79 (3H, *s*)	16.8
**27**	0.81 (3H, *s*)	14.8	0.84 (3H, *s*)	25.8
**28**		181.3	1.10 (3H, *s*)	27.8
**29**	0.99 (3H, *s*)	29.1	0.99 (3H, *s*)	28.0
**30**	1.02 (3H, *s*)	30.4	0.98 (3H, *s*)	15.3
**C=O CH$_3$**			0.79 (3H, *s*)	

Myricetin-3-*O*-β-galactopyranoside (3) and Rutin (4)

Table S2: ^{1}H (400 MHz) and ^{13}C (100 MHz) NMR spectral data of **C3** and **C4** in $CDOD_3$

	**C3**		**C4**	
**No.**	**δ_H (*mult, J*)**	**δ_C**	**δ_H (*mult, J*)**	**δ_C**
**2**		157.2		157.1
**3**		134.5		134.3
**4**		177.9		178.0
**5**		161.6		161.5
**6**	6.2 (*d*, *J* =2.08 Hz)	98.4	6.23 (*d*, *J* = 2.04 Hz)	98.6
**7**		164.6		164.6
**8**	6.39 (*d*, *J* = 1.92 Hz)	93.2	6.42 (*d*, *J* = 2.04 Hz)	93.5
**9**		156.9		158.0
**10**		105.7		104.2
**1`**		120.2		121.7
**2`**	7.38 *s*)	108.5	6.89 (*d*, *J* = 8.5 Hz)	114.7
**3`**		144.9		144.3
**4`**		136.7		148.4
**5`**		144.9	7.69 (*d*, *J* = 2.1 Hz)	116.3
**6`**	7.38 (*s*)	108.5	7.65 (*dd*, *J* = 8.4, 2.1 Hz)	122.2
	**galactopyranoside**		**glucose**	
**1``**	5.21 (*d*, *J* = 7.8 Hz)	104.1	5.09 (*d*, *J* = 7.6 Hz)	103.6
**2``**	3.82	71.2	3.52	74.2
**3``**		73.2	3.44	76.7
**4``**	3.87 (*d*, *J* = 3.36 Hz)	68.6	3.29	69.9
**5``**	3.49	75.8		75.7
**6``**		60.5		67.1
			**rhamnose**	
**1```**			4.54 (*d*, *J* = 1.2 Hz)	101.0
**2```**				70.6
**3```**			3.55 (*d*, *J* = 2.3 Hz)	70.8
**4```**			3.30	72.5
**5```**			3.46 (*d*, *J* = 2.4 Hz)	68.3
**6```**			1.15 (*d*, *J* = 6.2 Hz)	17.0

Quercetin (5)

Table S3**:** ^{1}H (400 MHz) and ^{13}C (100 MHz) NMR spectral data of compound **C5** in CD_3OD

No.	δ_H (*mult, J*)	δ_C
**2**		147.4
**3**		135.8
**4**		175.9
**5**		161.1
**6**	6.08 (*d*, *J* = 2.04 Hz)	97.8
**7**		164.2
**8**	6.29 (*d*, *J* = 2.12 Hz)	92.9
**9**		156.8
**10**		103.1
**1`**		122.7
**2`**	7.63 (*d*, *J* = 2.16 Hz)	114.8
**3`**		144.8
**4`**		146.6
**5`**	6.79 (*d*, *J* = 8.48 Hz)	114.6
**6`**	7.54 (*dd*, *J* = 2.16, 8.48 Hz)	120.2
**5-OH**	12.18, (*brs*)	

Apigenin (6)

Table S4: ^{1}H (400 MHz) and ^{13}C (100 MHz) NMR spectral data of compound **C6** in DMSO

**No.**	**δ_H (*mult, J*)**	**δ_C**	**δ_C (Lit.** (Owen *et al.*, 2003))
**2**		162.0	165.30
**3**		104.5	103.78
**4**		183.4	183.07
**5**		165.9	166.46
**6**	6.1 (*d*, *J* = 1.72 Hz)	99.8	99.71
**7**		155.2	159.6
**8**	6.2 (*d*, *J* = 1.8 Hz)	94.4	93.99
**9**		154.5	157.33
**10**		109.5	103.6
**1`**		121.1	121.17
**2`**	7.6 (*d*, *J* = 8.84 Hz)	129.1	128.48
**3`**	7.2 (*d*, *J* = 8.60 Hz)	116.4	115.97
**4`**		161.7	162.9
**5`**	7.2 (*d*, *J* = 8.60 Hz)	116.4	115.97
**6`**	7.6 (*d*, *J* = 8.84 Hz)	129.1	128.48
**5-OH**	12.6 (*s*)		

Amentoflavone (7)

Table S5**:** ^{1}H (400 MHz) and ^{13}C (100 MHz) NMR spectral data of compound **C7** in pyridine

	**Unit I**		**Unit II**	
**No.**	**δH (*mult, J*)**	**δC**	**δH (*mult, J*)**	**δC**
**2**		166.1		162.7
**3**		102.4		102.4
**4**		183.2		183.5
**5**		163.02		162.7
**6**	6.65 (*d*, *J* = 2.04 Hz)	100.3	6.89 (*d*, *J* = 8.8 Hz)	99.8
**7**		165.6		165.4
**8**	6.54 (*d*, *J* = 2.04 Hz)	94.6		103.9
**9**		155.7		154.5
**10**		109.4		109.4
**1`**		121.4		120.5
**2`**		129.1	7.88 (*d*, *J* = 8.8 Hz)	129.1
**3`**	7.14 (*d*, *J* = 8.8 Hz)	116.8	7.32 (*d*, *J* = 8.56 Hz)	116.2
**4`**		161.2		159.1
**5`**		120.1	7.32 (*d*, *J* = 8.65 Hz)	116.2
**6`**	7.65 (*d*, *J* = 8.56 Hz)	132.4	7.88 (*d*, *J* = 8.8 Hz)	129.1

Quercetin-3-*O*-β-glucoside (8)

Table S6: ^{1}H (400 MHz) and ^{13}C (100 MHz) NMR spectral data of compound **C8** in CD_3OD

No.	δ_H (*mult, J*)	δ_C	δ_C (Lit. (Zhang *et al.*, 2014))
**2**		157.05	156.79
**3**		134.36	133.99
**4**		178.14	177.97
**5**		161.61	161.71
**6**	6.23 (*d*, *J* = 2.04 Hz)	98.53	99.16
**7**		164.74	164.66
**8**	6.42 (*d*, *J* = 2.04 Hz)	93.34	93.98
**9**		157.40	156.79
**10**		104.22	104.39
**1`**		121.46	121.59
**2`**	7.86 (*d*, *J* = 2.16 Hz)	116.39	115.67
**3`**		144.42	145.30
**4`**		148.56	148.95
**5`**	6.89 (*d*, *J* = 8.52 Hz)	114.69	116.44
**6`**	7.62 (*dd*, *J* = 2.16, 8.52 Hz)	121.53	122.46
**1``**	5.17 (*d*, *J* = 7.76 Hz)	103.99	102.33
**2``**	3.58 (*m*)	71.77	71.70
**3``**	3.84 (*m*)	73.68	73.69
**4``**	3.87 (*m*)	68.60	68.41
**5``**	3.49 (*m*)	75.77	76.32
**6``**	3.65 (*m*)	60.52	60.62

α -Amyrin (9), β-Amyrin (10), and Lupeol (11)

Table S7: **:** ^{1}H NMR (400 MHz) and ^{13}C NMR (100 MHz) of compounds **C9**, **C10**, and **C11** in $CDCl_3$

	**C9**		**C10**		**C11**	
**No.**	**δ_H (*mult, J*)**	**δ_C**	**δ_H (*mult, J*)**	**δ_C**	**δ_H (*mult, J*)**	**δ_C**
**1**		38.8		38.7		38.6
**2**		28.7		27.2		27.5
**3**	3.22 (*dd*, *J* = 5.2, 10.8 Hz)	79.06	3.21 (*dd*, *J* = 4.8, 10.5 Hz)	79.03	3.17(*d*, *J* = 5.1 Hz)	79.0
**4**		38.7		38.5		39.8
**5**	0. 76	55.3	0.72	55.3	0.69	55.1
**6**		18.4		18.6		19.0
**7**		32.2		32.4		34.3
**8**		40.7		39.8		41.7
**9**		47.7		47.6		50.7
**10**		36.6		36.9		37.2
**11**		23.3		23.6		21.2
**12**	5.13 (*t*, *J* = 3.6 Hz)	124.4	5.19 (*t*, *J* = 3.5 Hz)	121.7		25.3
**13**		139.6		145.2		38.6
**14**		42.1		41.7		42.8
**15**		27.2		26.2		27.2
**16**		26.6		26.1		35.9
**17**		33.7		32.6		43.0
**18**	1.31	59.1	1.54	47.8	2.39	48.3
**19**		39.6	1.92	47.3		47.7
**20**		39.6		31.0		150.9
**21**		31.2		34.7		30.1
**22**	1.85	41.5		37.1		40.8
**23**	0.83	28.1	0.77	28.0	0.80	28.7
**24**	0.76	15.6	0.90	15.5	0.77	15.7
**25**	0.73	15.6	0.73	15.4	0.84	16.2
**26**	0.83	16.9	0.93	16.1	1.04	16.1
**27**	1.01	23.2	1.19	25.9	0.96	14.5
**28**	0.94	28.1	1.07	28.4	0.80	18.1
**29**	0.79	17.9	0.87	33.8	4.69 (*s*)<br>4.56 (*s*)	109.3
**30**	0.86	19.4	0.80	23.7	1.70	19.8

ANNEXURE ONE

NMR SPECTRA OF COMPOUNDS ISOLATED FROM *RHUS LUCIDA*

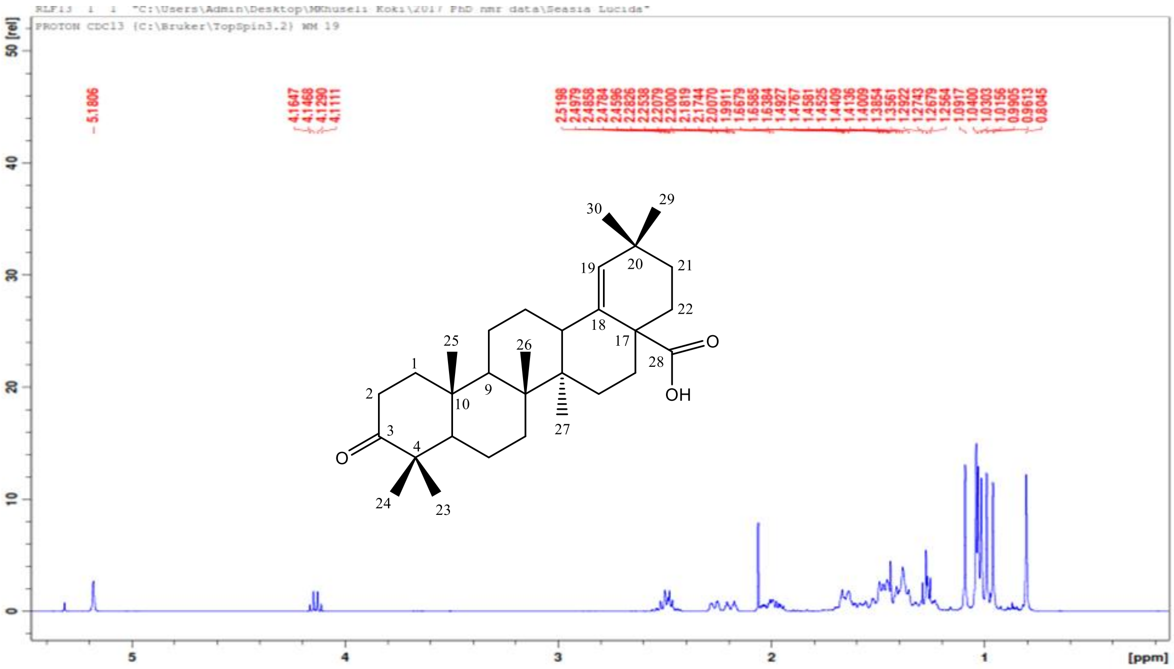

Figure S1: ^{1}H NMR moronic acid (C1) in $CDCl_3$

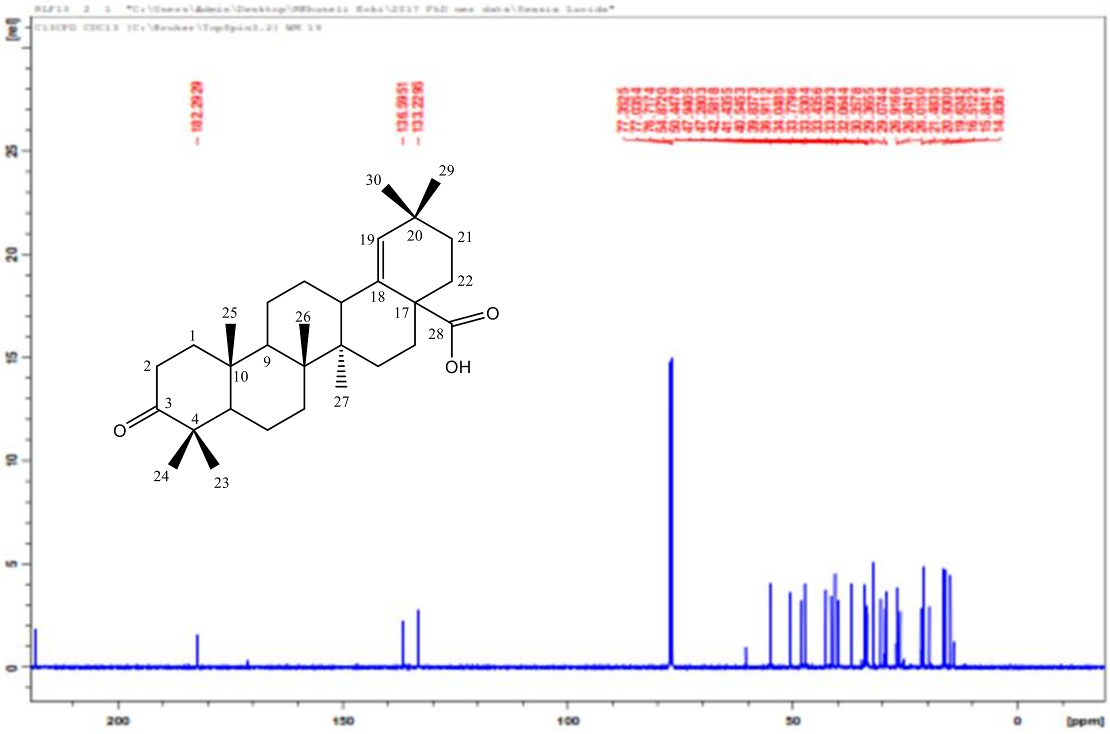

Figure S2: ^{13}C NMR of moronic acid (C1) in $CDCl_3$

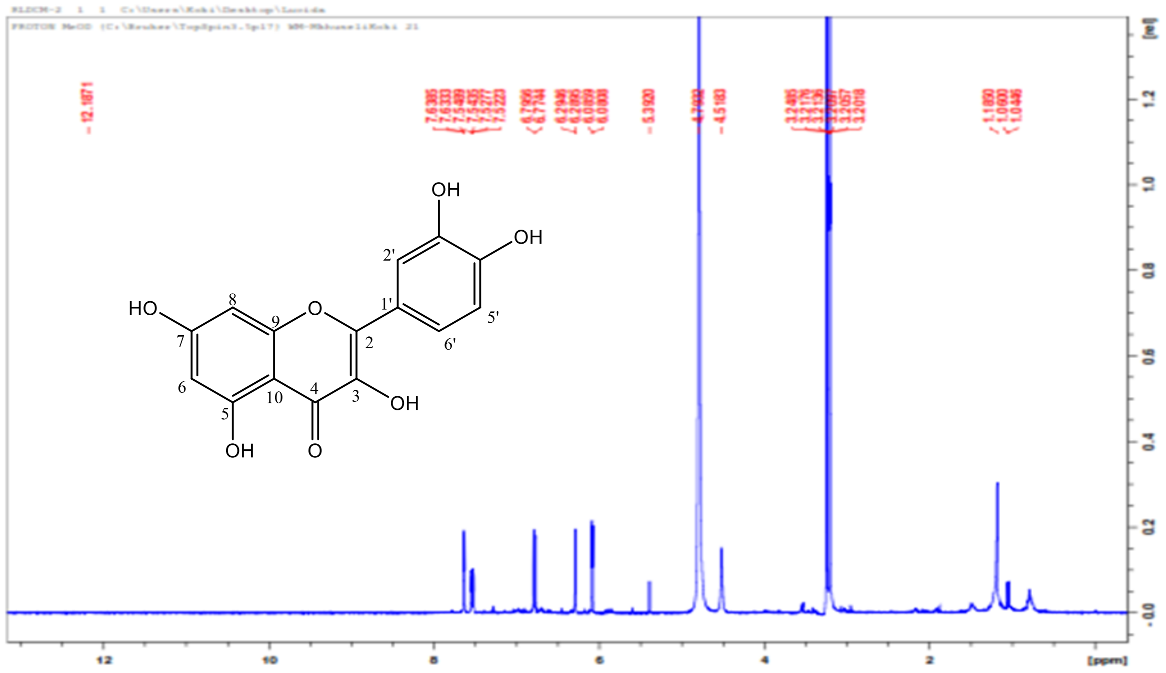

Figure S3: ^{1}H NMR of quercetin (C5) in CD_3OD

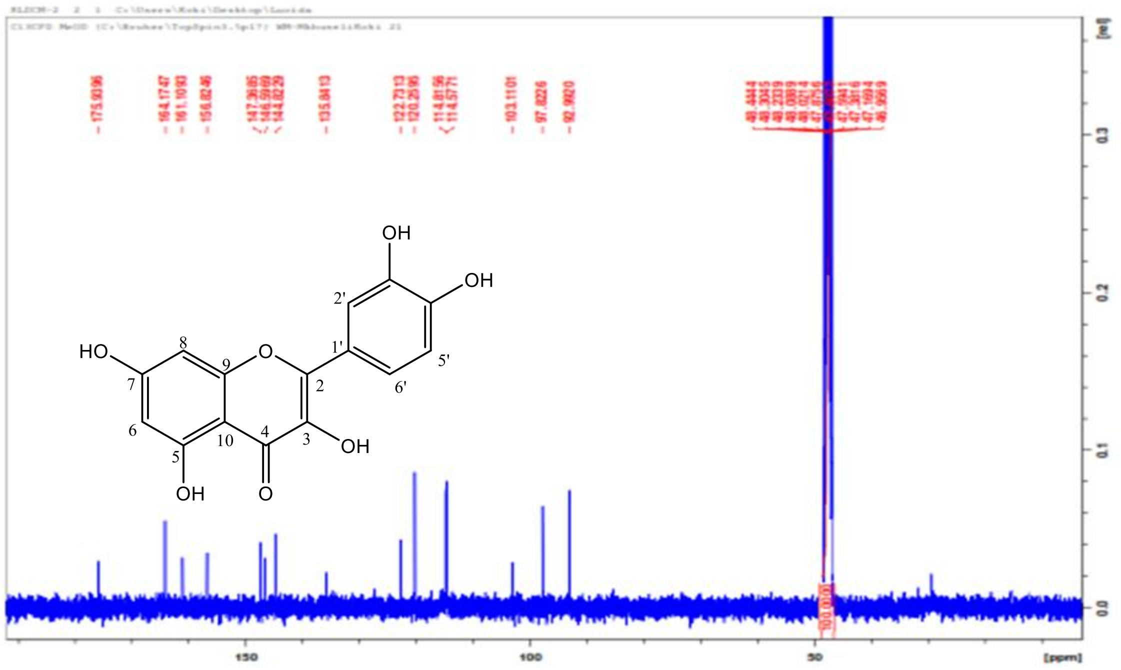

Figure S4: ^{13}C NMR of quercetin C5 in CD_3OD

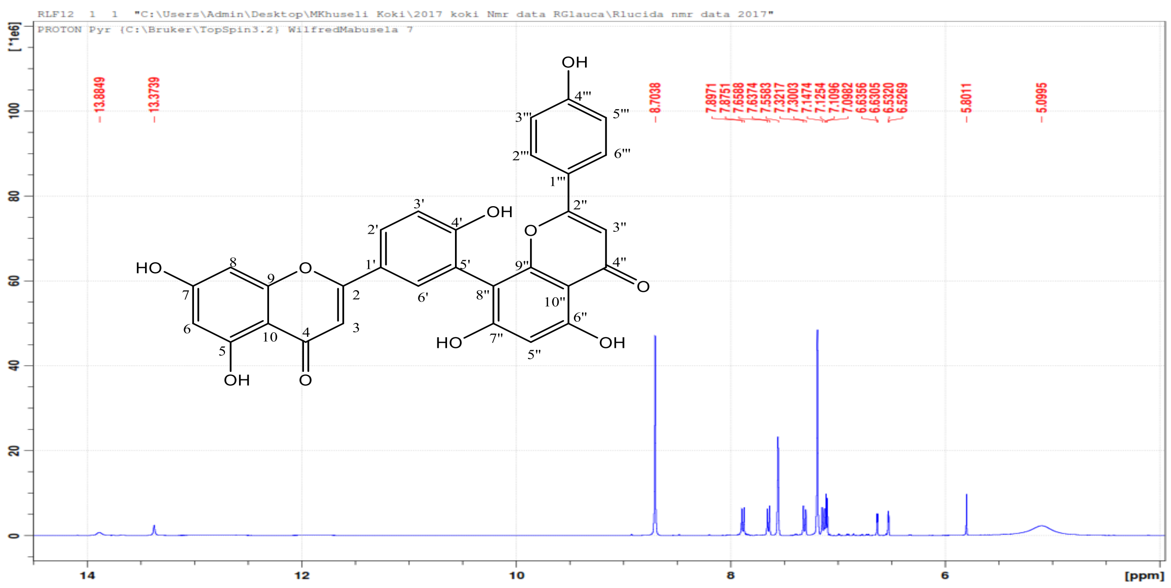

Figure S5: ^{1}H NMR of amentoflavone (C7) in Pyridine-d_5

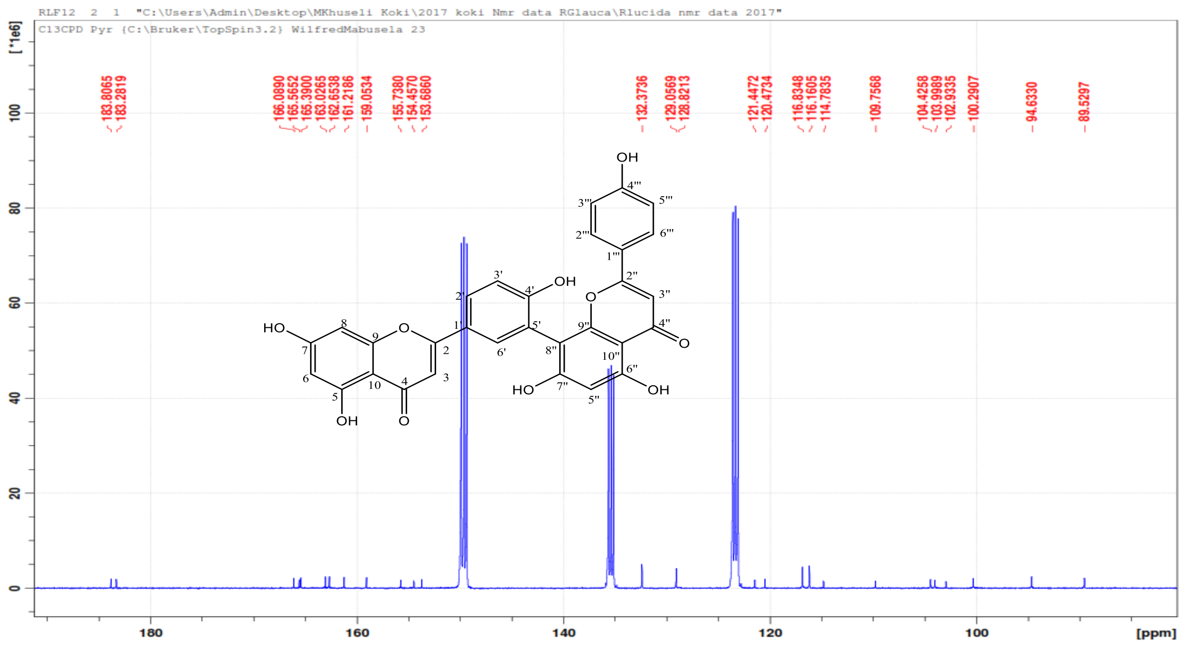

Figure S6: ^{13}C NMR of amentoflavone (C7) in Pyridine-d_5

ANNEXURE TWO

NMR SPECTRA OF COMPOUNDS ISOLATED FROM *R. GLAUCA*

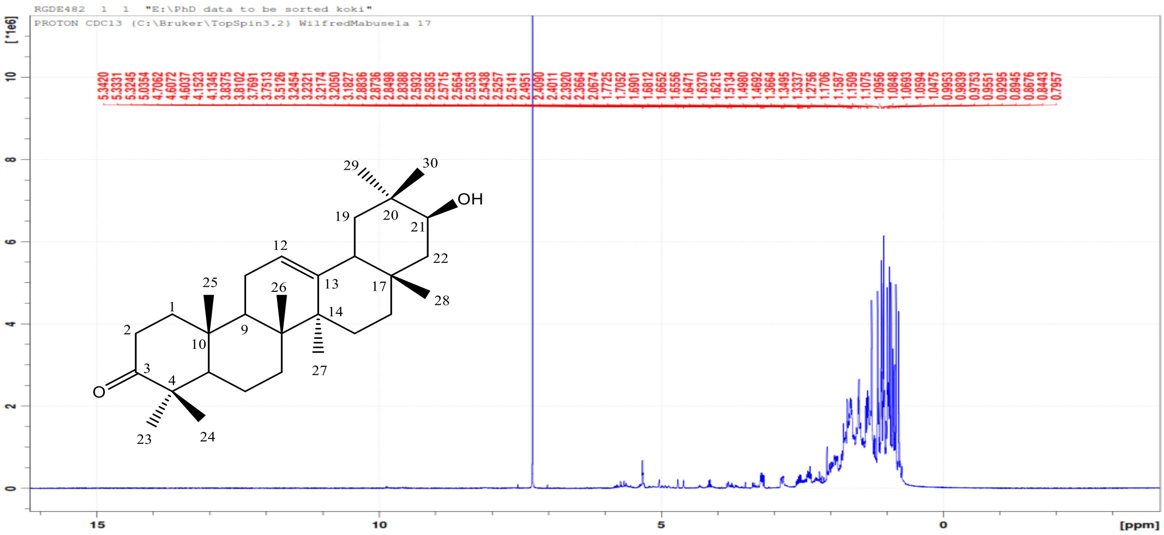

Figure S7: ^{1}H NMR spectrum of 21-*β*-hydroxylolean-12-en-3-one (C2) in $CDCl_3$

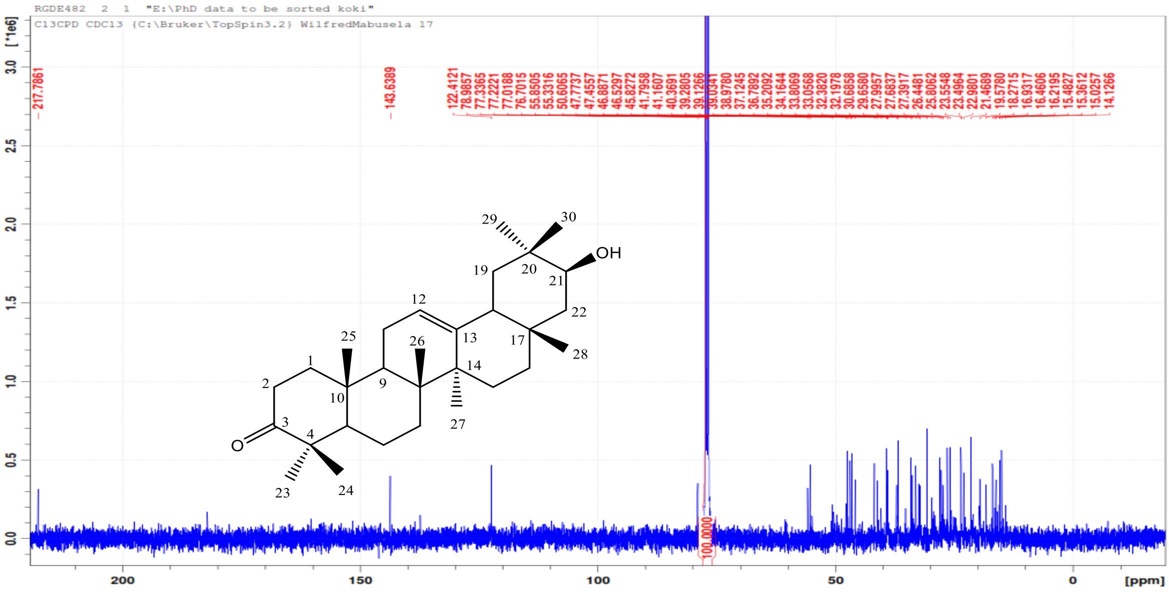

Figure S8: ^{13}C NMR spectrum of 21-*β*-hydroxylolean-12-en-3-one (C2) in $CDCl_3$

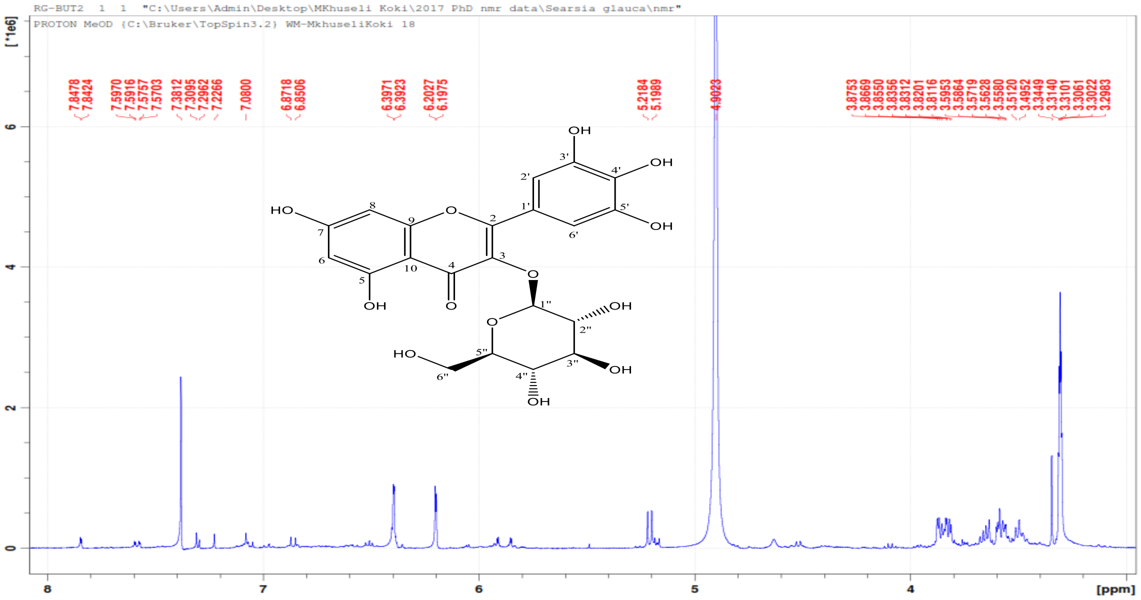

Figure S9: ^{1}H-NMR of myricetin 3-*O*-*β*-galactopyranoside (C3) in CD_3OD

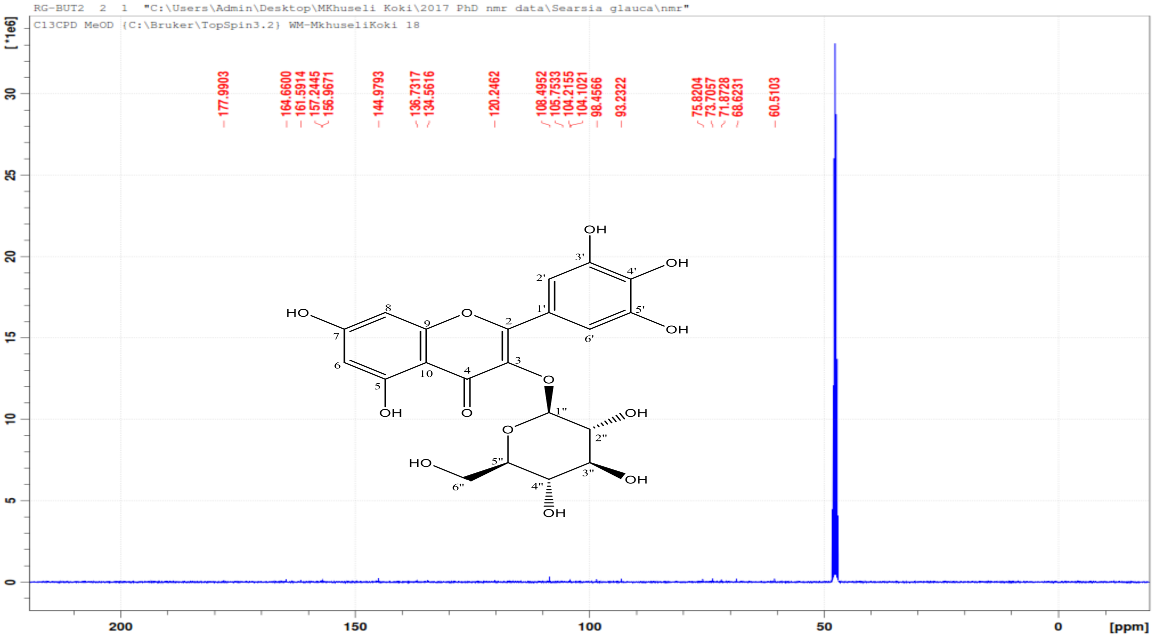

Figure S10: ^{13}C-NMR of myricetin 3-*O*-*β*-galactopyranoside (C3) in CD_3OD

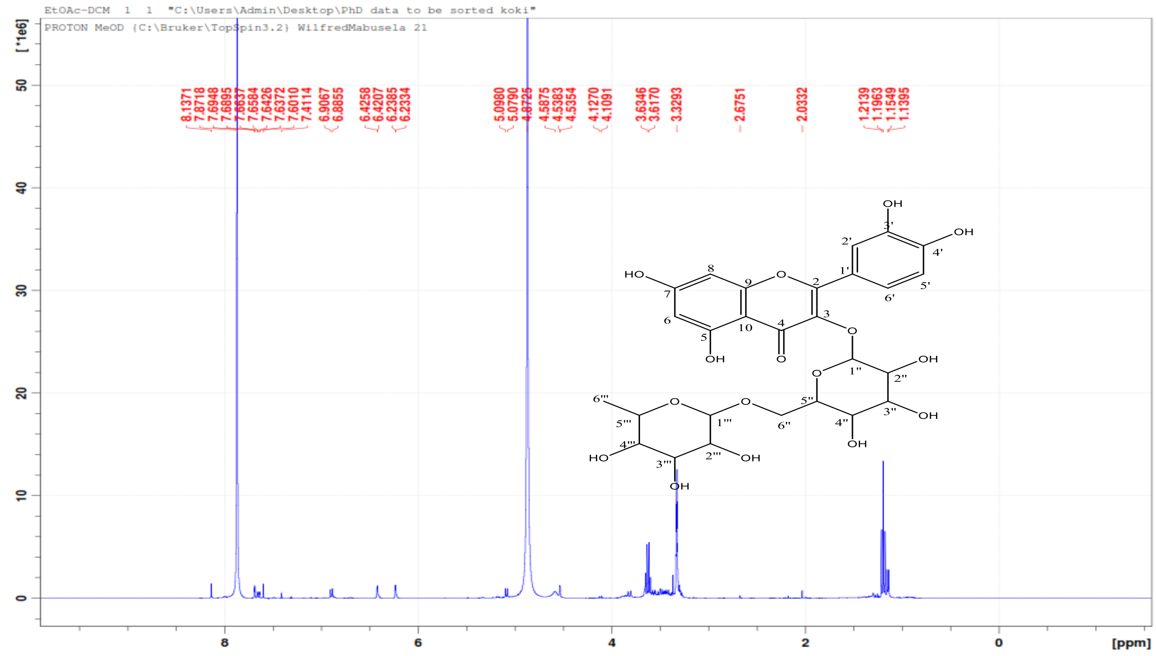

Figure S11: ^{1}H NMR of Rutin (C4) in CD_3OD

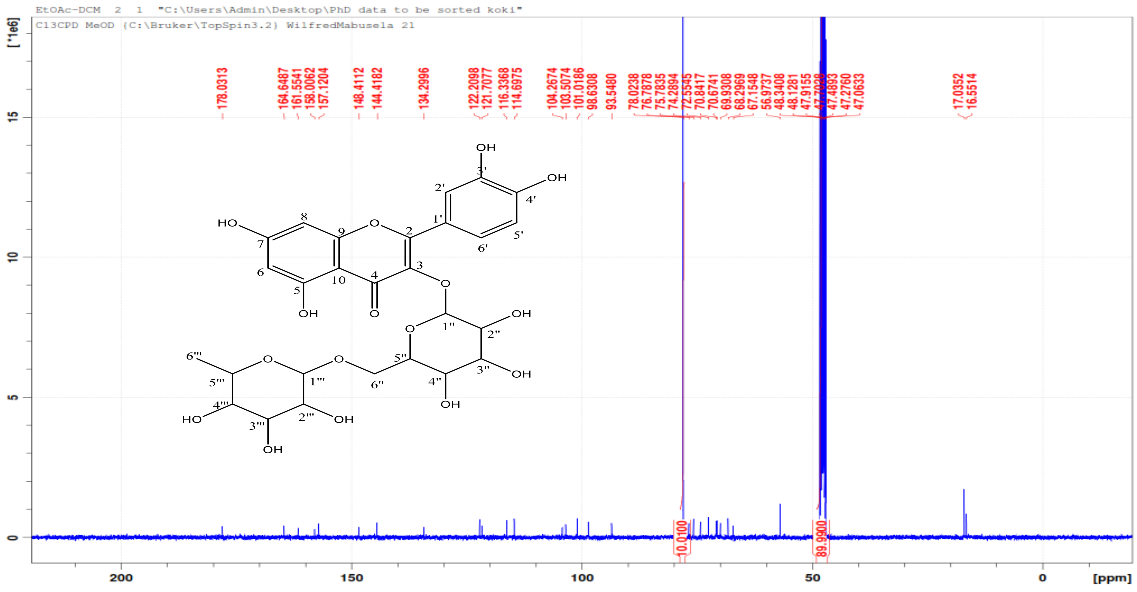

Figure S12: ^{13}C NMR of Rutin (C4) in CD_3OD

ANNEXURE THREE

NMR SPECTRA OF COMPOUNDS ISOLATED FROM *R. LAEVIGATA*

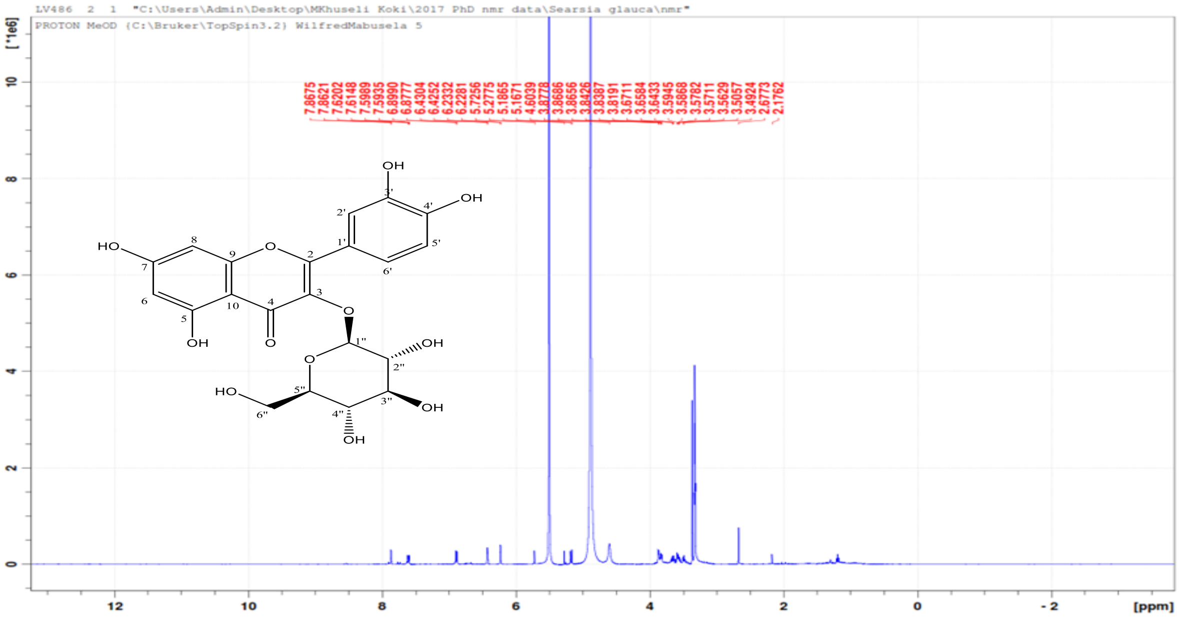

Figure S13: ^{1}H NMR of Quercetin-3-*O*-*β*-glucoside (C8) in CD_3OD

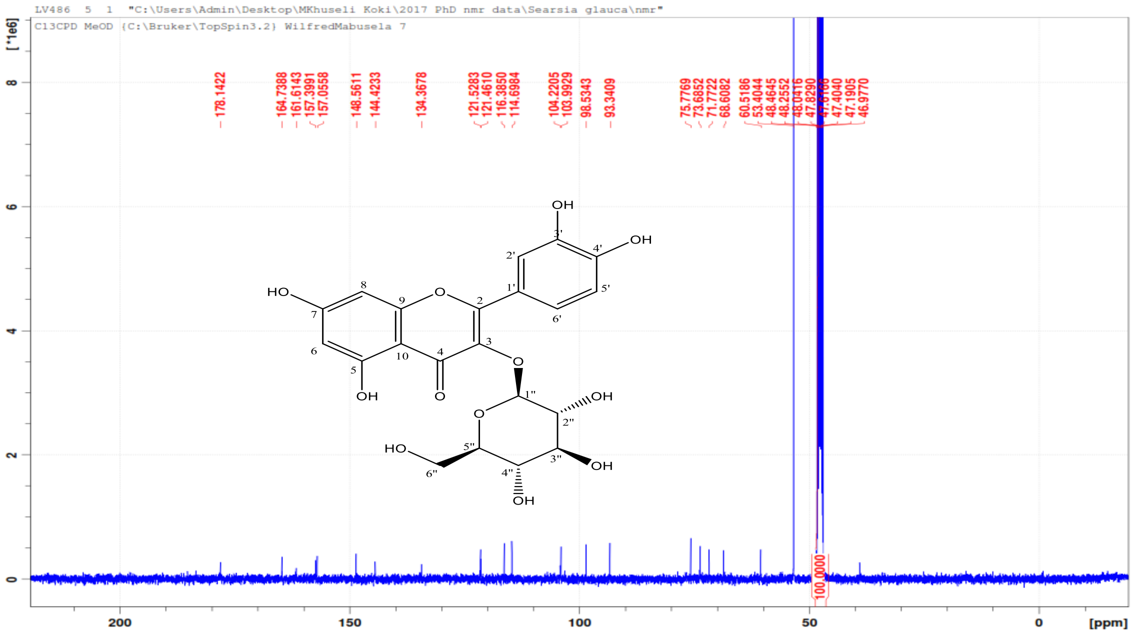

Figure S14: ^{13}C NMR of Quercetin-3-*O*-*β*-glucoside (C8) in CD_3OD
